# Inhibition of MARCH5 ubiquitin ligase abrogates MCL1-dependent resistance to BH3 mimetics *via* NOXA

**DOI:** 10.18632/oncotarget.7558

**Published:** 2016-02-21

**Authors:** Aishwarya Subramanian, Adrian Andronache, Yao-Cheng Li, Mark Wade

**Affiliations:** ^1^ Center for Genomic Science of IIT@SEMM, Fondazione Istituto Italiano di Tecnologia, Milano, Italy; ^2^ Gene Expression Laboratory, Salk Institute for Biological Studies, La Jolla, CA, USA

**Keywords:** MARCH5, mitochondria, MCL1, NOXA, ubiquitin

## Abstract

BH3 mimetic compounds induce tumor cell death through targeted inhibition of anti-apoptotic BCL2 proteins. Resistance to one such compound, ABT-737, is due to increased levels of anti-apoptotic MCL1. Using chemical and genetic approaches, we show that resistance to ABT-737 is abrogated by inhibition of the mitochondrial RING E3 ligase, MARCH5. Mechanistically, this is due to increased expression of pro-apoptotic BCL2 family member, NOXA, and is associated with MARCH5 regulation of MCL1 ubiquitylation and stability in a NOXA-dependent manner. *MARCH5* expression contributed to an 8-gene signature that correlates with sensitivity to the preclinical BH3 mimetic, navitoclax. Furthermore, we observed a synthetic lethal interaction between MCL1 and MARCH5 in MCL1-dependent breast cancer cells. Our data uncover a novel level at which the BCL2 family is regulated; furthermore, they suggest targeting MARCH5-dependent signaling will be an effective strategy for treatment of BH3 mimetic-resistant tumors, even in the presence of high MCL1.

## INTRODUCTION

Mitochondria-dependent apoptosis is governed by the activities of the BCL2 family. Essentially, the death-inducing activities of pro-apoptotic BH3 members BAX and BAK are negatively regulated by several anti-apoptotic family members, including BCL2, BCL2L1 (BCLXL), and MCL1 [1]. Molecular targeting of this pathway has demonstrated that drugging protein-protein interactions can be therapeutically beneficial. Specifically, treatment of several cancer types with the ABT-737 compound (or its clinical derivative, navitoclax/ABT-263) leads to tumor cell death and tumor regression in pre-clinical and clinical studies [2, 3].

These compounds are known as BH3 mimetics, as they recapitulate an alpha-helical structure of BH3-only proteins that mediates binding to anti-apoptotic BCL2 proteins. Thus, BH3 mimetics act as competitive inhibitors that unleash pro-apoptotic proteins from their negative regulators. Despite their efficacy in a number of cell lines, neither ABT-737 nor navitoclax inhibit MCL1 [4]. This is clinically relevant, since MCL1 is overexpressed in many tumors, including those originating in lymphoid, breast, and colon tissues. Several MCL1 inhibitors have been reported [5], yet direct experimental evidence of their efficacy and selectivity for MCL1 in cell-based studies is scarce [5-7]. A notable exception is the indole-2-carboxylic acid core-based MCL1 inhibitor, A-1210477, which has greatly improved binding affinity for MCL1, and exhibits on-target activity in several MCL1-dependent cell lines [8, 9]. Despite these advances, no MCL1 inhibitors have yet reached the clinical testing stage. Thus, alternative strategies for improving the efficacy of current BH3 mimetics in tumors with high levels of MCL1 are required.

In addition to their key role in metabolism and apoptosis, mitochondria are active nodes in many signaling networks. The organelles send and receive signals *via* many outer membrane-associated proteins, including kinases and ubiquitin ligases [10]. Mitochondrial-associated ubiquitin ligases play clear roles in mitochondrial function and apoptosis in neurodegenerative disease [11, 12]. However, much less is understood regarding their role in cancer. During the course of our studies, we became interested in the MARCH (for *m*embrane-*a*ssociated *R*ING-*CH*) ligase family. Primarily known for their immunomodulatory roles [13], each of the 11 MARCH family members has specialized and unique functions [14]. Among them, MARCH5 is the only mitochondria-localized member; several studies have thus focused on its role as a regulator of mitochondrial morphology, particularly with regard to neurodegeneration [15-18]. Very recently, MARCH5-dependent suppression of ERK signaling was implicated in the maintenance of ES cell pluripotency [19].

Here, we investigated whether MARCH5 was a regulator of mitochondria-driven apoptosis in cancer cells. Strikingly, we found that loss of MARCH5 sensitizes to ABT-737 treatment in a BAX-dependent, but BAK-independent manner. Knockdown of either MARCH5 or MCL1 sensitized cells to ABT-737, suggesting these proteins are part of a shared apoptotic network. Paradoxically, however, there was a robust upregulation of MCL1 following MARCH5 loss, despite the increased sensitivity to ABT-737. This is since MARCH5 regulates MCL1 stability in a NOXA-dependent (but PUMA and BIM-independent) manner. The sensitization to ABT-737 is also dependent on NOXA, and partially dependent on the p53 tumor suppressor protein. Together, our data uncover a novel link between MARCH5 and MCL1, and also suggest an additional strategy for abrogating MCL1-dependent resistance to BH3 mimetics.

## RESULTS

### MARCH5 knockdown sensitizes cells to BH3-mimetics, yet stabilizes MCL1

**Figure 1:**
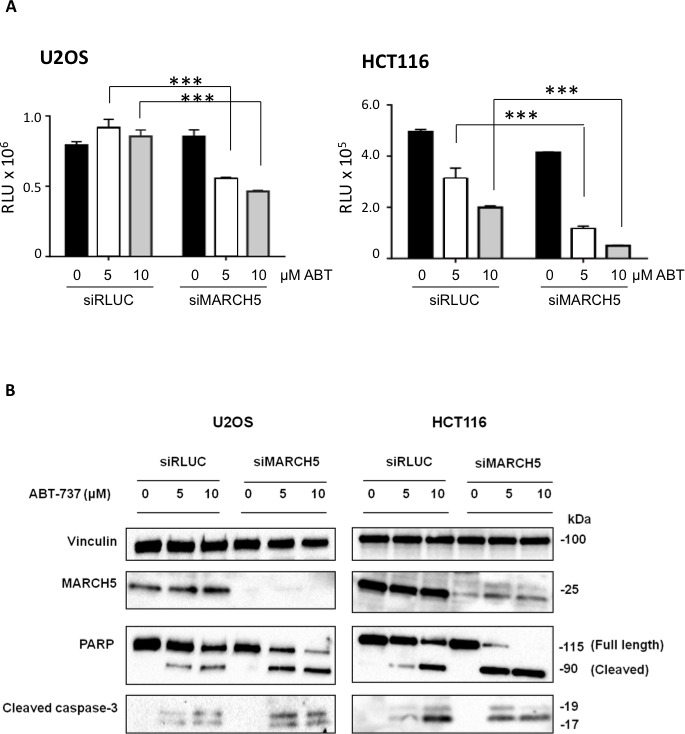
MARCH5 depletion sensitizes cell lines to BH3-mimetic induced apoptosis **A.** U2OS and HCT116 cells transfected with siRNA targeting MARCH5 or a control siRNA targeting luciferase were treated for 24 h with ABT-737 at the indicated concentrations. Viability was measured with Cell Titer Glo. Error bars are standard deviation from triplicate experiments. The asterisks (***) indicate a *p* value of < 0.001 compared to the respective controls using Student's unpaired *t*-test. **B.** Lysates from U2OS and HCT116 cells transfected with siRLUC or siMARCH5 and treated with ABT-737 at the indicated concentrations for 24 h were subjected to SDS-PAGE and western blotting.

Ubiquitylation events at the mitochondrial outer membrane surface are key drivers of apoptosis. Due to its subcellular localization and enzymatic activity, therefore, we reasoned that MARCH5 might regulate cell death. To test this, we depleted *MARCH5* with a pool of 4 siRNAs prior to treatment with the BH3 mimetic, ABT-737. Figure 1A shows that MARCH5 knockdown sensitized cells to the compound, and that the mode of death was apoptosis, as indicated by cleavage of caspase-3 to its active form, and cleavage of PARP, a caspase substrate (Figure 1B; for quantification of PARP cleavage, see Figure S1). Several independent siRNAs and C911 controls confirmed that the sensitization was on-target (Figure S2).

**Figure 2:**
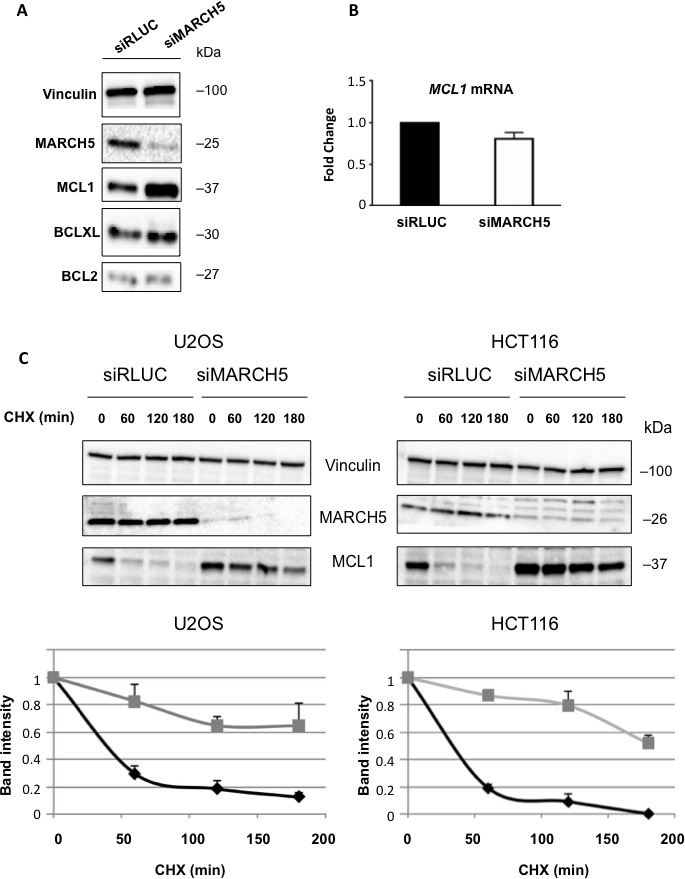
Loss of MARCH5 leads to stabilization of MCL1 **A.** Lysates from HCT116 cells transfected with control siRNA (siRLUC) or siRNA targeting MARCH5 were subjected to SDS-PAGE and western blotting. **B.** *MCL1* mRNA levels were measured following MARCH5 knockdown using quantitative RT-PCR and were normalized to *18S* mRNA. Error bars indicate the SD of triplicate measurements. **C.** Cycloheximide pulse-chase experiments were performed by treating transfected cells with cycloheximide for the given time points. Lysates were subjected to SDS-PAGE and immunoblot analysis to observe MCL1 stability. Western blot panels are representative of three independent experiments. Graphs show the MCL1 protein band intensities normalized to the loading control. Black diamonds, siRLUC; gray squares, siMARCH5. Error bars are standard deviation of triplicate experiments.

Although ABT-737 can effectively antagonize BCL2, BCL2L1(BCLXL), and BCL2L2(BCLW), it is unable to antagonize MCL1. This presents a significant barrier to efficacy of ABT-737 in the clinic, as many tumors overexpress the latter protein. Given that both MARCH5 and MCL1 knockdown elicit the same phenotype [4, 20] (i.e., sensitization to ABT-737) we hypothesized that loss of MARCH5 might be accompanied by a reduction in MCL1. Strikingly, however, we observed the exact opposite: knockdown of MARCH5 engendered a robust increase in MCL1 levels, despite the clear sensitization to ABT-737 (Figure 2A). This effect was selective, as levels of other anti-apoptotic BCL2 members did not change upon MARCH5 loss (Figure 2A; Figure S2 shows effect was ‘on-target’). *MCL1* mRNA was not increased following loss of MARCH5, but MCL1 protein half-life was significantly longer (Figure 2B, 2C). Together, these data show that MCL1 is stabilized at the post-translational level after MARCH5 knockdown.

### p53, BAX, and NOXA contribute to sensitization following loss of MARCH5

**Figure 3:**
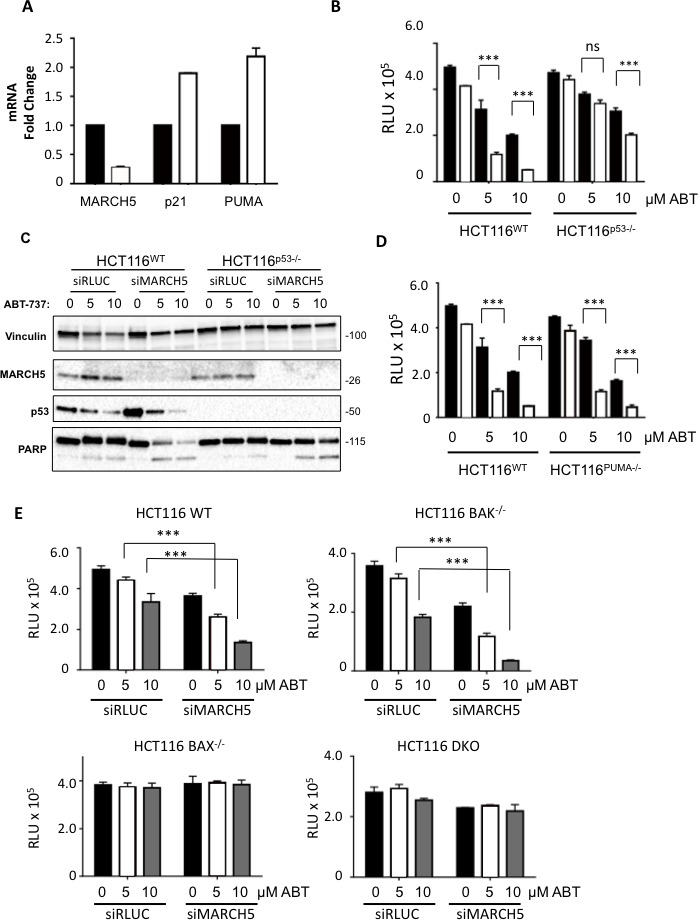
MARCH5 depletion upregulates p53 transcriptional targets and sensitizes cells to p53- and BAX-dependent apoptosis **A.** The expression of *MARCH5* mRNA, and the p53 targets-*PUMA* and *p21*, following control siRNA (black bars) or MARCH5 knockdown (white bars) in HCT116 cells were assessed by quantitative RT-PCR. Error bars indicate the SD of triplicate measurements. **B.** Isogenic HCT116^WT^ and HCT116^p53−/−^ cells were transfected with control siRNA (black bars) or siRNA targeting MARCH5 (white bars) and treated with ABT-737 at the indicated concentrations. Cellular viability was measured using the CellTiter Glo assay. Error bars indicate the SD of triplicate experiments. **C.** HCT116^WT^ and HCT116^p53−/−^ cells were transfected with siRLUC or siMARCH5 and treated with ABT-737 at the indicated concentrations. Whole cell lysates were harvested and subjected to SDS-PAGE and western blot analysis. **D.** Isogenic HCT116^WT^ and HCT116PUMA−/− cells were transfected with control siRNA (black bars) or siRNA targeting MARCH5 (white bars) and treated with ABT-737 at the indicated concentrations for 24 h. Cellular viability was measured using the CellTiter Glo assay. **E.** WT, *BAK−/−*, *BAX−/−* and *BAK/BAX* DKO HCT116 cells were depleted of MARCH5 and treated with ABT-737 at the given concentrations. Error bars are standard deviation. For all graphs, the asterisks (***) indicate a *p* value of < 0.001 and “ns” indicates no significant difference compared to the respective controls using Student's unpaired *t*-test.

We first focused on p53, as several of its downstream transcriptional targets are activated upon ABT-737 treatment, and p53 activation synergizes with BH3 mimetics [21]. Indeed, p53 and several of its target genes were upregulated in MARCH5-knockdown cells compared to controls (Figure 3A, 3C). Furthermore, experiments with isogenic HCT116-p53^WT^ and HCT116-p53^NULL^ cells revealed that the sensitization to ABT-737 was partially p53-dependent (Figure 3B, 3C). However, the enhanced death we observed did not require PUMA, a BH3 pro-apoptotic p53 transcriptional target (Figures 3D, S1D and [22]). We also examined the requirement for both BAX (another p53 target) and BAK (a pro-apoptotic family member that is predominantly inhibited in cells by MCL1 [23]). Isogenic cell lines revealed that sensitization was BAX-dependent, but BAK-independent (Figure 3E). Together our results show that a PUMA-independent, BAX-dependent apoptotic signaling pathway is primed upon loss of MARCH5, and sensitizes cells to ABT-737 independently of MCL1 levels.

**Figure 4:**
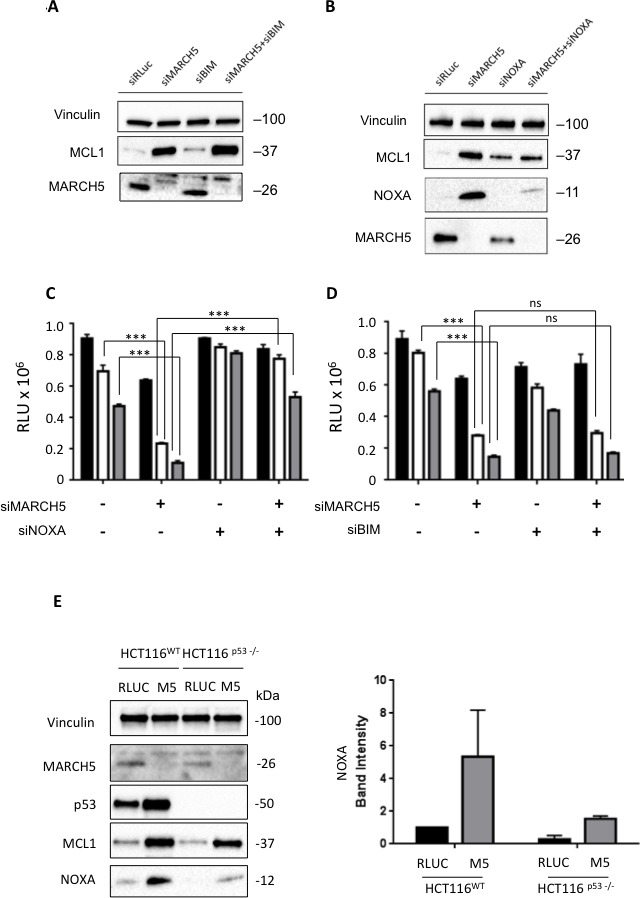
Sensitization to apoptosis and MCL1 stabilization upon MARCH5 loss are NOXA-dependent **A.** Lysates from HCT116 cells transfected with the indicated siRNAs were subjected to SDS-PAGE and western blotting. Due to poor antibody immunoreactivity, validation of BIM knockdown was performed using qPCR (Figure S7). **B.** Lysates from HCT116 cells transfected with the indicated siRNAs were subjected to SDS-PAGE and western blotting. **C.** HCT116 cells transfected with siRLUC, siMARCH5, siNOXA or co-transfected with siMARCH5/siNOXA were treated with DMSO (black bars) or ABT-737 (5 or 10 μM, white or gray bars, respectively). **D.** HCT116 cells transfected with siRLUC, siMARCH5, siBIM or co-transfected with siMARCH5/siBIM were treated with DMSO (black bars) or ABT-737 (5 or 10 μM, white or gray bars, respectively). Error bars are standard deviation from 3 independent experiments. Validation of knockdown for panels **C.** and **D.** was performed by quantitative RT-PCR, and mRNA levels were normalized to *18S* mRNA (Figure S7). **E.** HCT116^WT^ and HCT116^p53−/−^ cells were transfected with control siRNA or siRNA targeting MARCH5. Lysates were then subjected to SDS-PAGE and western blotting. NOXA induction was quantified from triplicate independent experiments.

At first glance, the increased sensitivity to ABT-737 in the presence of increased MCL1 is paradoxical. We thus hypothesized that one of the pro-apoptotic BH3 proteins might neutralize MCL1's pro-survival activity. The two main candidates for this role are BIM and NOXA [24-26]. Following MARCH5 knockdown, BIM loss had no effect on MCL1 levels (Figure 4A). Strikingly, however, NOXA was concomitantly upregulated with MCL1 after MARCH5 knockdown. Consistent with previous reports [27], knockdown of NOXA alone engendered slight upregulation of MCL1. However, NOXA loss also robustly attenuated the induction of MCL1 that we observed upon MARCH5 knockdown (Figure 4B). Together, these data indicate that NOXA is required for maximal stabilization of MCL1 following loss of MARCH5. These data are consistent with other reports of NOXA-dependent stabilization of MCL1 [28, 29].

We then tested whether NOXA was also required for MARCH5-dependent sensitization to ABT-737. Double knockdown experiments revealed that loss of NOXA (but not BIM) abrogated sensitization (Figures 4C and 4D, S1E and S1F). Since NOXA is a p53 transcriptional target [30], we examined whether MARCH5-dependent upregulation of NOXA required p53. NOXA steady state levels also increased in HCT116-p53^NULL^ cells after MARCH5 knockdown, although the absolute level was lower than observed in HCT116-p53^WT^ cells (Figure 4E). These findings were also confirmed using esiRNA (Figure S3), strongly suggesting that NOXA-dependent sensitization was on-target. These data thus reveal a novel, p53-independent mode of NOXA upregulation in response to MARCH5 loss.

### MARCH5 regulation of MCL1 ubiquitylation and stability requires NOXA

**Figure 5:**
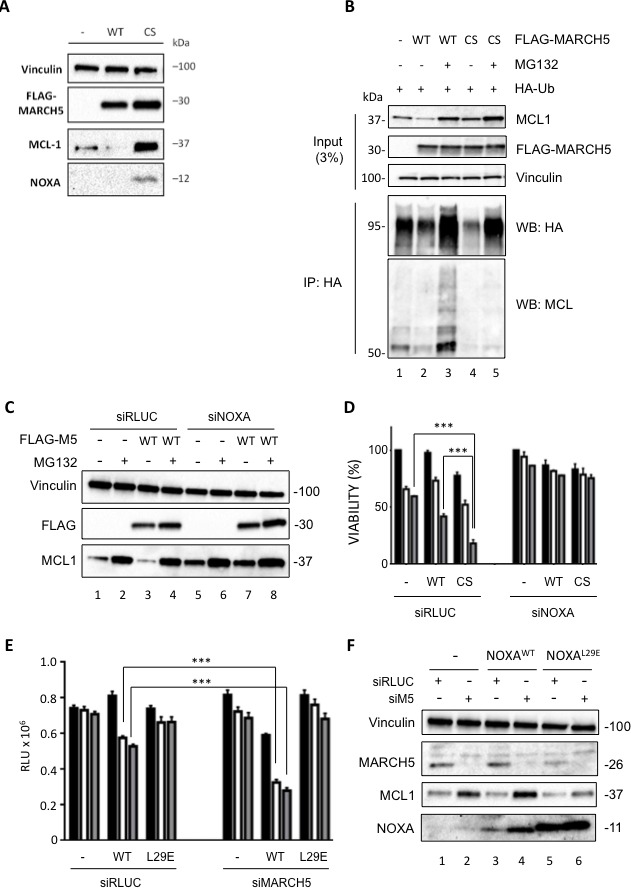
A MARCH5 RING domain mutant stabilizes MCL1 and NOXA, and sensitizes cells to ABT-737 **A.** U2OS cells stably expressing Dox-inducible FLAG-MARCH5^WT^ or FLAG-MARCH5^C65S68S^ (CS) were induced with 50 ng/ml Dox for 24 h. Whole cell lysates were then subjected to SDS-PAGE and western blotting. **B.** Whole cell lysates from U2OS cells stably expressing Dox-inducible FLAG-MARCH5^WT^ or FLAG- MARCH5^CS^ transfected with the indicated plasmids and treated with Doxycyline (50 ng/ml) and/or MG132 (10 μM) as shown were subjected to immunoprecipitation using anti-HA antibody. Immunoprecipitated complexes were then subjected to SDS-PAGE and immunoblotted with the indicated antibodies. Input blots represent levels of indicated proteins in the whole cell lysate. **C.** U2OS cells stably expressing Dox-inducible FLAG-MARCH5^WT^ were transfected with control siRNA or siRNA targeting NOXA. Twenty-four hours post-transfection, cells were treated with 50 ng/ml Dox for a further 24 h to induce the expression of FLAG-MARCH5. Cells expressing FLAG-MARCH5^WT^ were also treated with MG132 for 3 h to block the proteasomal degradation of MCL1. Lysates were subjected to SDS-PAGE and western blotting. Knockdown of *NOXA* mRNA in U2OS FLAG-MARCH5^WT^ was assessed by quantitative RT-PCR (Figure S7). Error bars indicate the SD of triplicate measurements. **D.** U2OS cells stably expressing Dox-inducible FLAG-MARCH5^WT^ or FLAG-MARCH5^CS^ were transfected with control siRNA or siRNA targeting NOXA. Cells were treated 24 h post-transfection with 50 ng/ml Dox to induce the expression of MARCH5 constructs, then treated for 24 h with DMSO (black bars) or ABT-737 (5 or 10 μM, white or gray bars, respectively). Error bars are standard deviation. **E.** HeLa cells stably expressing Dox-inducible NOXA^WT^ or NOXA^L29E^ were transfected with control siRNA or siRNA targeting MARCH5. Six hours post transfection, cells were treated with 50 ng/ml Dox for 48 h to induce the expression of NOXA constructs. Cells were then seeded on 96-well plates and treated with DMSO (black bars) or ABT-737 (5 or 10 μM, white or gray bars, respectively) for 24 h. Cell viability was assessed using CellTiter Glo. (***, *p* < 0.001; unpaired t test). **F.** Whole cell lysates from cells treated as in **E.** were subjected to SDS-PAGE and western blot analysis.

To complement the genetic studies, we performed cell-based assays using doxycycline (Dox)-inducible MARCH5^WT^ and a ligase-deficient mutant, MARCH5^CS^. Overexpression of MARCH5^WT^ reduced endogenous MCL1 levels, whereas MARCH5^CS^ stabilized both MCL1 and NOXA (Figure 5A). Compared with MARCH5^WT^, less ubiquitylated MCL1 was co-immunoprecipitated with HA-ubiquitin following MARCH5^CS^ overexpression, even in the presence of proteasome inhibitor (compare lanes 3 and 5, Figure 5B). These data indicate that MCL1 ubiquitylation is controlled by MARCH5 ligase activity, and that the MARCH5^CS^ mutant stabilizes MCL1 by preventing its ubiquitylation. Strikingly, knockdown of NOXA abrogated MARCH5-dependent downregulation of MCL1 (Figure 5C, compare lanes 1 and 3 with lanes 5 and 7). Together, these data demonstrate that MARCH5-dependent regulation of MCL1 stability requires NOXA. We also examined whether MARCH5^CS^ altered the cellular response to ABT-737. Figure 5D shows that expression of Dox-inducible MARCH5^CS^ reduced viability compared to both uninduced and Dox-inducible MARCH5^WT^ cells. Furthermore, MARCH5^CS^ sensitized cells to ABT-737-induced apoptosis (Figure 5D), and this was inhibited upon knockdown of NOXA. This is consistent with our MARCH5 siRNA data, and indicates that inhibition of MARCH5 ligase activity is sufficient to activate NOXA-dependent sensitization.

### NOXA/MCL1 binding is important for sensitization upon MARCH5 loss

The above data suggest that endogenous NOXA is required for both MCL1 stabilization and sensitization to ABT-737 upon MARCH5 loss. However, they do not show that a direct functional interaction between NOXA and MCL1 is required for this phenotype. To test this, we knocked down MARCH5 in the presence of either wild type NOXA, or an MCL1 binding-deficient NOXA mutant (L29E) [31]. Interestingly, NOXA^WT^ was not toxic in the absence of ABT, but became so when MARCH5 was concomitantly knocked down (Figure 5E), and this correlated with increased levels of NOXA (Figure 5F, compare lanes 3 and 4). Furthermore, MARCH5-dependent sensitization to ABT-737 was enhanced following induction of NOXA^WT^, but was completely abrogated in the presence of NOXA^L29E^ (Figure 5E). Importantly, the MCL1 stabilization accompanying MARCH5 loss was also attenuated in the presence of NOXA^L29E^ (Figure 5F, compare lanes 5 and 6 with lanes 3 and 4). This phenocopies the results of the NOXA siRNA experiments above (Figure 4). Together, these data support the results of our NOXA siRNA experiments, and extend them by revealing a direct role for NOXA in the inactivation of MCL1 following loss of MARCH5.

### MARCH5 contributes to a gene signature associated with ABT-263 sensitivity

Several factors in addition to MCL1 are associated with sensitivity to BH3 mimetics [32-35], and our data suggested that MARCH5 may also contribute to the response to ABT-737. We therefore performed a multiple linear regression analysis (see Supplemental Experimental Procedures and Table S1) to determine whether *MARCH5* expression (alone or in combination with selected other factors) was predictive of sensitivity to ABT-263 (the orally bioavailable version of ABT-737, also known as navitoclax). We stratified the groups based on p53 status, since it is known that p53 modulates BH3 mimetic-induced death.

**Figure 6:**
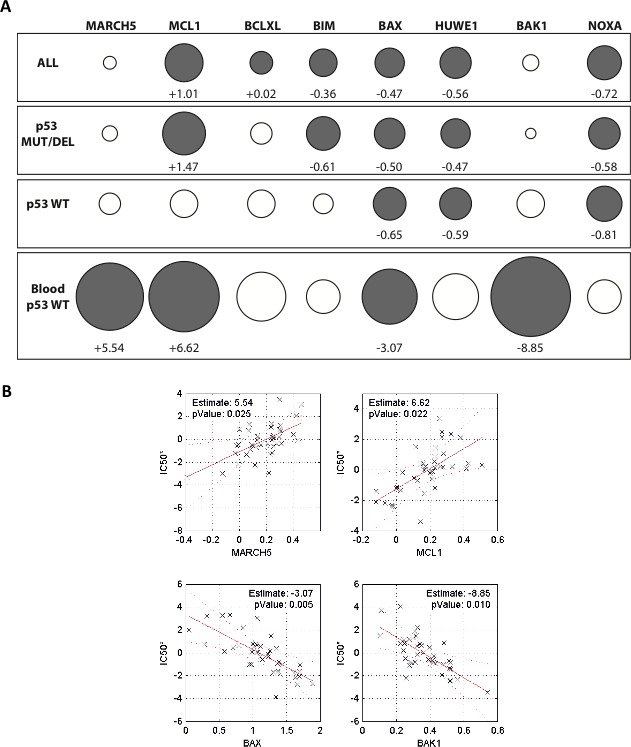
Expression of MARCH5 and MCL1 are significant contributors to a gene signature predicting sensitivity to ABT-263/navitoclax **A.** Contribution of 8 genes to a prediction of navitoclax sensitivity based on mRNA expression across a panel of 648 cell lines (221 wild type p53, 427 non-functional p53). Circles (gray closed circles *p* < 0.05; white open circles, ns) depict ‘Effect’ sizes, with values shown for significant genes only. ‘Effect’ is defined as the unit change in navitoclax IC50 value per unit increase in gene expression (thus positive Effect values reduce sensitivity and negative Effect values increase sensitivity). The size of the circle is proportional to the effect size. **B.** Relationship between navitoclax sensitivity and expression of the four genes that significantly contribute to the sensitivity profile of the ‘Blood p53 WT’ subset in panel A. Each datapoint represents one cell line. Values on the y-axis (IC50^*^) are regression-adjusted IC50 values. Values on the x-axis are regression-adjusted mRNA expression levels. Estimate values are derived from slope of the regression line and reflect the effect size. For example, as MARCH5 expression increases in cell lines (upper left panel), the navitoclax IC50^*^ increases, indicating a reduced sensitivity to the drug. All numerical values and a complete description of the multiple regression analysis can be found in Supplementary Table S1 and Supplemental Information.

Figure 6A shows the results of the optimized multiple linear regression model. Consistent with their known influence on ABT-263 sensitivity, expression of BAX, HUWE1, and NOXA were significant contributors to the gene signature in cells expressing either wild type or nonfunctional (mutated or deleted) p53. Interestingly, MCL1 expression was a strong determinant of the ABT-263 sensitivity of cells expressing nonfunctional p53. By contrast, the effect of MCL1 in cells with wild type p53 was much weaker. This underscores the finding that the level of MCL1 expression alone is not always sufficient to predict ABT-737 sensitivity [34, 36], and also suggests that other p53-induced factors can to some extent attenuate the anti-apoptotic function of MCL1 [37]. Although our biological data above show that MARCH5 is a clear modulator of the response to ABT-737, its expression was not identified as a significant determinant of sensitivity in the multiple regression analysis of the ‘ALL’ cell line group. Since this group is composed of cell lines from diverse tumor types, we reasoned that re-analysis based on tissue of origin might provide further insight. We therefore focused on comparing data from all wild type p53 cell lines to the data from the hematological malignancies (‘Blood’) subset. This choice was based on evidence from both cell-based and preclinical animal models that individuals with liquid tumors comprise a suitable target population for BH3 mimetic treatment. As expected, the pro-apoptotic effectors BAX and BAK were associated with increased ABT-263 sensitivity in these malignancies (Figure 6A). Strikingly, increased expression of both MARCH5 and MCL1 were significant determinants of ABT-263 sensitivity in the blood subset (Figure 6A, lower panel). Figure 6B is a graphical representation of the contribution of these four genes to ABT-263 sensitivity, clearly indicating that MARCH5 and MCL1 expression levels were inversely correlated with ABT-263 sensitivity, whereas increased expression of BAX and BAK was associated with increased sensitivity. Together with our current data, this analysis suggests that MARCH5 is a context-dependent modulator of the sensitivity to BH3 mimetics.

### MARCH5-dependent chemical and genetic synthetic lethalities

**Figure 7:**
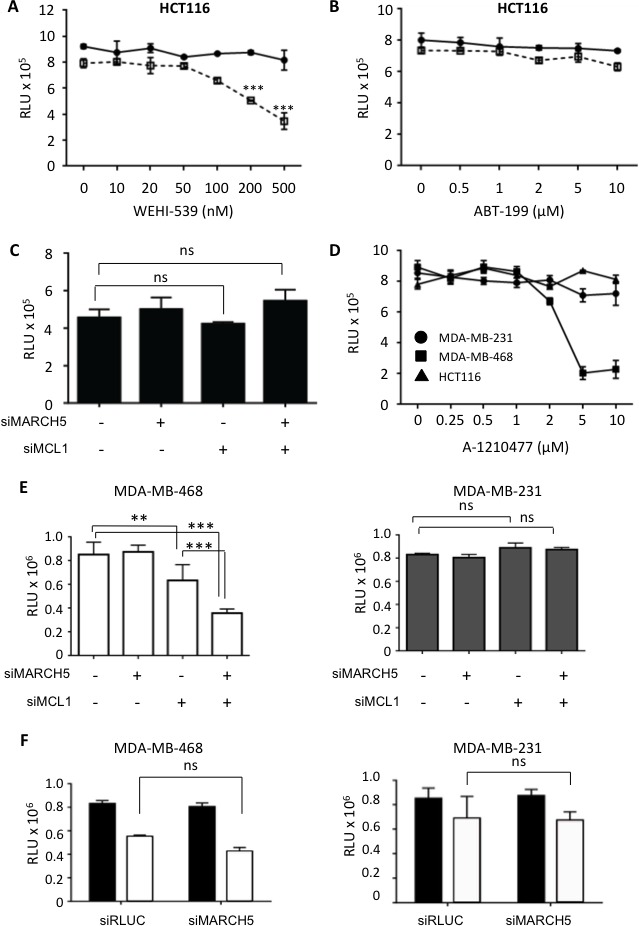
MARCH5 loss is synthetic lethal with the BCLXL inhibitor, WEHI-539, and with MCL1 depletion in MCL1-driven breast cancer lines HCT116 cells transfected with siRLUC (closed circles) or siMARCH5 (open squares) were treated with WEHI-539 **A.** or ABT-199 **B.** at the indicated doses. **C.** HCT116 cells were depleted of MARCH5, MCL1, or both, and viability was assessed with Cell Titer Glo. **D.** HCT116, MDA-MB-231, and MDA-MB-468 cells were treated with the MCL1 specific inhibitor, A1210477 at the indicated concentrations and viability was assessed with Cell Titer Glo. **E.** MDA-MB-231 and MDA-MB-468 cells were depleted of MARCH5 or MCL1, or both. Validation of knockdown was performed by quantitative RT-PCR and mRNA levels were normalized to *18S* mRNA (Figure S7). **F.** MDA-MB-231 and MDA-MB-468 cells were transfected with control siRNA or siRNA targeting MARCH5 and treated with DMSO (black bars) or 10 μM ABT-737 (white bars). Error bars are standard deviation from at least 3 replicate experiments

The above studies demonstrate a MARCH5-dependent chemical-genetic lethal interaction with the BCL2 pathway. Since ABT-737 inhibits BCL2, BCLXL, and BCLW, it cannot be used to determine the relative contribution of each anti-apoptotic protein to the sensitization we observed. To address this, we exploited more specific BCL2 antagonists that were developed during the course of our studies [38, 39]. We observed synthetic lethality in HCT116 following MARCH5 knockdown in the presence of the BCLXL-selective antagonist, WEHI-539, but not with ABT-199, a BCL2-selective antagonist (Figure 7A, 7B). Together, these data indicate that cells rely upon BCLXL for survival when MARCH5 is inactivated. This is consistent with a predominant role for BCLXL, rather than BCL2, in protecting cells in which MCL1 has been functionally inactivated [35].

Given the MARCH5-MCL1 link we discovered, we next investigated whether there was a synthetic lethal relationship between these two genes in cancer cells. In HCT116, this was not the case (Figure 7C). However, chemical and genetic inhibition of MCL1 in HCT116 revealed that they are not dependent on MCL1 for growth in these short-term assays (Figure 7C, 7D). This is consistent with the finding that BCLXL ensures survival of several cell types when MCL1 is compromised (our WEHI-539 data above and see [40]). We therefore hypothesized that MARCH5 loss may instead exhibit synthetic lethality in cell lines that are known to be MCL1-dependent. To test this, we selected triple-negative breast cancer cell lines that differ with regard to their dependence on MCL1 [34]. Using an MCL1-selective antagonist and MCL1 siRNA, we confirmed that MDA-MB-468 cells are MCL1-dependent, whereas MDA-MB-231 cells are MCL1-independent (Figure 7D, 7E and [9, 34]). Strikingly, concomitant loss of MARCH5 and MCL1 was synthetic lethal in MCL1-dependent MDA-MB-468 cells, but not in the MCL1-independent MDA-MB-231 line (Figure 7E). Both these cell lines harbor inactive p53 mutant proteins (http://p53.free.fr/Database/Cancer_cell_lines/Breast_cancer.html). Consistent with their p53 status, and with our results in isogenic HCT116-p53^WT^ and HCT116-p53^NULL^ cells (Figure 3B), loss of MARCH5 did not sensitize these cell types to ABT-737 (Figure 7F). Together, these data suggest that MARCH5 loss requires p53 for maximal sensitization to BH3 mimetics; however, factors beyond p53 (including MCL1 dependency as we show here) determine the intrinsic cellular sensitivity to depletion of MARCH5.

## DISCUSSION

At least four RING E3 ubiquitin ligases (MARCH5, RNF144B, TRIM59, and MULAN) are constitutively localized at the mitochondrial outer membrane (MOM) [41]. Here, we identify MARCH5 as a novel determinant of the sensitivity to ABT-737. Mechanistically, we found that a NOXA/MCL1 axis is activated following MARCH5 loss, and that NOXA is required for enhanced BH3-dependent apoptosis. This pathway is specifically activated by loss of MARCH5, as we did not observe increased levels of MCL1 following knockdown of the MOM E3 ligase, RNF144B. Furthermore, although RNF144B knockdown can sensitize cells to BAX-dependent apoptosis [42], it did not sensitize cells to ABT-737 (Figure S2A, S2B). Our results suggest that MOM E3 ligase profiling (and elucidation of their downstream signaling networks) may offer an additional strategy for inducing death in BH3 mimetic-resistant cancer cells.

Although our data suggest otherwise, it is possible that another MCL1 E3 ligase is *inactivated* following loss of MARCH5. FBXW7 isoforms have been implicated in regulation of MCL1 levels [43]. However, in contrast to MARCH5 knockdown, we observed only slight changes in MCL1 steady state levels following loss of FBXW7 (Figure S4). This is consistent with a previous report that FBXW7 does not regulate MCL1 levels in non-stressed conditions [44], and we therefore consider a MARCH5/FBXW7/MCL1 axis unlikely.

Previous studies of MARCH5 have focused on its role in mitochondrial dynamics [15, 45]. Indeed, MARCH5 is reported to modulate the levels or activity of the pro-fission DRP1 GTPase, and of the pro-fusion GTPases, MFN1 and -2 [46, 47]. We can exclude the possibility that a change in mitochondrial morphology per se elicits MCL1 stabilization. This is since treatment with the mitochondrial decoupling agent CCCP, which induced an identical perinuclear mitochondrial morphology to that observed following MARCH5 loss, did not stabilize MCL1. Additionally, treatment of cells with the DRP1 inhibitor, mdivi-1 [48], had no impact on the levels of MCL1 (Figure S5). Together with our BH3 profiling results, these data indicate that MCL1 stabilization upon MARCH5 depletion is mediated *via* regulation of NOXA, rather than by changes in mitochondrial morphology.

Our finding that NOXA and MCL1 are co-stabilized in ABT-sensitive cells was initially puzzling. However, both MARCH5-dependent sensitization and MCL1 stabilization were lost following co-depletion of NOXA. Additionally, sensitization to ABT-737 was abrogated by a NOXA mutant that exhibits attenuated MCL1 binding. We therefore suggest that following loss of MARCH5, NOXA is upregulated in order to bind to, and functionally inhibit, MCL1. This would provide a ‘decision point’ for entry into apoptosis, which is triggered either by a stoichiometric excess of NOXA over MCL1, or upon activation of additional pro-apoptotic BH3 proteins by compounds such as ABT-737. In support of this, concomitant NOXA and MCL1 upregulation is observed in MYC-driven leukemias [49], and following activation of RAS in epithelial cells [50]. Furthermore, despite concomitant upregulation of NOXA and MCL1, squamous cell carcinomas retain sensitivity to ABT-737 due to an increased NOXA:MCL1 ratio [36]. Treatment with only WEHI-539, a BCLXL-selective inhibitor, had no effect on cell viability, but was synthetic lethal with MARCH5 loss. This phenocopies the effect observed when MCL1-null cells are treated with WEHI-539, as cells in which MCL1 is functionally inactivated rely on BCLXL for survival [9, 39]. Together, these data provide further evidence that the concomitant upregulation of NOXA with MCL1 following MARCH5 loss is a ‘priming event’ for apoptosis, most likely due to functional inactivation of MCL1. These observations, together with our current results, suggest that inhibition of MARCH5 and activation of NOXA will be effective even in tumors with high MCL1 levels.

The functional interaction between MARCH5 and p53 is intriguing. Whereas p53 was required for maximal sensitization to ABT-737, it was dispensable for the synthetic lethal interaction between MARCH5 and MCL1. This is supported by our experiments with MDA-MB-468 and MDA-MB-231: both these lines carry mutant p53, and were not sensitized to ABT-737 by MARCH5 knockdown, yet MCL1/MARCH5 synthetic lethality was retained in MCL1-dependent MDA-MB-468. In NOXA, we have identified a key downstream p53 target that is involved in sensitization to ABT-737. However, the upstream signals that lead to p53 stabilization upon MARCH5 inhibition remain unclear.

Our data indicate that MARCH5 has an anti-apoptotic role in cancer cells. Furthermore, a survey of the COSMIC database indicates that *MARCH5* mRNA is overexpressed in a restricted set of tumor types (our unpublished observations). However, the frequency of MARCH5 mutations in primary tumor samples and in tumor cell lines is low (our unpublished observations). Thus, we infer that MARCH5 is among the growing number of ‘non-oncogenes’ that are nevertheless important for cancer cell survival during tumorigenesis or following treatment with therapeutic agents. Consistent with this hypothesis, we found that *MARCH5* expression contributed to an 8-gene index that correlated with sensitivity to ABT-263 (Navitoclax) in hematological malignancies. Indeed, its contribution was as significant as that of MCL1, a well-validated determinant of sensitivity to BH3 mimetics. Interestingly, our regression analysis also identified that BAK was an important determinant of ABT-263 sensitivity in the hematopoietic compartment (Figure 6A, lower panel). This engenders confidence in our regression analysis, as previous reports indicate that BAK (and to a lesser extent, BAX, as we also found here) is a significant effector of the intrinsic mitochondrial apoptosis pathway [51]. Due to low sample sizes in the publically available databases, we were unable to extend our analyses to other tissue types. Therefore, further *in vivo* studies with appropriate tumor models are now required to determine whether targeted inhibition of MARCH5 will have a therapeutic benefit in cancer. As with all cancer targets, this is likely to depend on the precise genetic makeup of individual tumors. Furthermore (as recently reported for MCL1 in the case of triple-negative breast cancer [34]), predictive signatures should additionally take into account not only mRNA expression, but also protein levels of MARCH5 and BCL2 family members.

Breakthroughs in the design of selective MCL1 inhibitors indicate that direct targeting of this oncogene to induce cell death is now possible. Current data indicate that these compounds are effective in MCL1-dependent tumors, but have variable results in other cell lines. Confirming this, we observed that treatment with A-1210477 alone induced death in MCL1-dependent breast cancer cells, but not in HCT116 cells, which are not dependent on MCL1 (Figure 7). Combined treatment with high dose ABT-737 and A-1210477 induced cell death in HCT116 (Figure S6), but in this case was no more effective than a combination of ABT-737 and MARCH5 knockdown. Together, these data clearly indicate that targeting MARCH5 will be particularly effective in combination with broad spectrum BH3 mimetics, or with the next generation of MCL1-selective antagonists. Direct inhibition of RING E3 ligases remains an important yet challenging therapeutic goal; whether MARCH5 will be amenable to such an approach remains to be seen. Elucidation of MARCH5 substrates and the identification of factors that control MARCH5 levels and activity in tumor cells will suggest potential strategies that can be exploited in order to inhibit this mitochondrial ligase.

## MATERIALS AND METHODS

### Cell culture

Cells were grown in McCoy's 5A (HCT116), DMEM (U2OS, MDA-MB-231 and HeLa), or DMEM/HAM's F12 (MDA-MB-468) media supplemented with 10% fetal bovine serum, 4 mM L-glutamine, 100 U/ml penicillin, and 100 U/ml of streptomycin, in a humidified atmosphere of 5% CO_2_ at 37°C. Cells were passaged prior to reaching full confluency for general maintenance. DMEM, EMEM, L-glutamine, penicillin and streptomycin were purchased from Lonza (Basel, Switzerland). McCoy's 5A and HAM's F12 were purchased from Gibco (ThermoFisher Scientific, MA, USA). Cells were purchased from ATCC (Manassas, VA). HCT116 p53^−/−^, BAX^−/−^, BAK^−/−^ and BAX/BAK^−/−^ were a kind gift from Dr. Bert Vogelstein, Johns Hopkins University. Doxycycline-inducible HeLa cells expressing either NOXA^WT^ or NOXA ^L29E^ were a kind gift from Dr. Andreas Villunger, Biocenter, Innsbruck Medical University.

### Compound treatments

Cells were allowed to attach overnight and then treated with indicated concentrations of compounds. All the solutions were adjusted to have an equivalent amount of DMSO (final DMSO not more than 0.1% in all experiments). Nutlin-3a was purchased from Sigma-Aldrich (St. Louis, MO, USA), ABT-737 was from Santa Cruz (Dallas, TX, USA), ABT-199 from Selleckchem (Houston, Texas, USA), and WEHI-539 (BCLXL inhibitor) and A1210477 (MCL1 inhibitor) were purchased from Chemietek (Indianapolis, IN, USA). MG-132 was purchased from Enzo Life Sciences (Farmingdale, NY, USA) and doxycycline, cycloheximide, CCCP, and mdivi-1 from Sigma Aldrich (St. Louis, MO, USA). For cycloheximide pulse chase assays, cells were then treated with cycloheximide (100 μg/ml) for the given time points before being subjected to western blot analysis.

### Plasmids, expression constructs and mutagenesis

Wild type human MARCH5 with a 3× N-terminal FLAG tag (a kind gift of Professor Shigehisa Hirose, Tokyo Institute of Technology) was subcloned into pLi196, a Dox-responsive entry vector for RMCE.[52] Using this plasmid as a template, the MARCH5 RING domain mutant MARCH5^CS^ (in which two Zn^2+^ co-ordinating cysteine residues (Cys65 and -68) in the RING domain are mutated to serine) was generated by site-directed mutagenesis (Stratagene). The primers used for the mutagenesis were as follows: Forward- 5′-attcagcattgctctgaggacttgccactctggctgtac-3′; Reverse- 5′-gtacagccagagtggcaagtcctcagagcaatgctgaat-3′. For transfection, U2OS cells were plated on 100-mm plates and transfected with 5 μg of empty vector or HA-ubiquitin (a kind gift from Dr. Simona Polo) with Lipofectamine 3000 reagent (Life Technologies, Carlsbad, CA, USA). Media was changed 6 h post-transfection and cells were harvested after 48 h.

### Generation of cell lines

All Dox-inducible FLAG-MARCH5 cell lines were created by recombination mediated cassette exchange (RMCE) of Dox-responsive FLAG-MARCH5 plasmids into a master parental U2OS cell line as previously described. [52, 53] Cells were induced with Dox (50 ng/ml) for 24 h to induce expression of the various FLAG-MARCH5 constructs before harvesting for western blot analysis. For RNAi experiments, cells were transfected with siRNA for 24 h and then induced with Dox for a further 24 h.

### RNAi experiments

siGENOME SMARTpool siRNAs for MARCH5, *Renilla* luciferase negative control, PLK1 positive control, MCL1, BIM, NOXA, and RNF144B were purchased from GE Life Sciences/Dharmacon (Lafayette, CO, USA). U2OS cells were seeded on 6-well plates for forward transfection and 25 nM siRNA was transfected with 3 μl DharmaFECT 1 (Dharmacon); HCT116 cells, MDA-MB-231, MDA-MB-468 and cells were transfected with 3 μl RNAiMAX (Life Technologies). Cells were harvested 48 h post-transfection for protein and RNA extraction or seeded on 96-well plates 24 h post-transfection and treated with compounds the following day for viability assays. Deconvolution experiments were performed with siGENOME individual siRNAs as well as C911 controls. siRNA sequences are in Supplemental Experimental Procedures, and validation of knockdown by qPCR for all genes is in Figure S7.

### Western blots and antibodies

Cells were lyzed in 0.5% NP-40 lysis buffer (50 mM Tris, pH 8.0, 5 mM EDTA, 150 mM NaCl, 0.5% NP-40, 1 mM phenylmethylsulfonyl fluoride, 1 mM sodium vanadate, 10 mM NaF and Complete Mini Protease Inhibitors (Roche, Nutley, NJ, USA), at 4°C for 30 min. Following SDS-PAGE electrophoresis, proteins were transferred onto nitrocellulose membranes. Membranes were incubated with the following antibodies: anti-MARCH5 (gifted by Dr. Nakamura, Tokyo Institute of Technology), anti-PARP (BD Biosciences), anti-MCL1 (Santa Cruz Biotechnologies), anti-PUMA (Santa Cruz Biotechnologies), anti-Actin (Sigma-Aldrich), anti-Vinculin (Sigma-Aldrich) anti-NOXA (Calbiochem), anti-p53 (Santa Cruz Biotechnologies), anti-BCL2 (BD Biosciences) anti-BCLXL (Cell Signaling Technologies), anti-cleaved caspase 3 (Cell Signaling Technologies). Rabbit and mouse secondary antibodies were purchased from Bio-Rad Laboratories. Blots were developed using Clarity Western ECL Substrate, Bio-Rad Laboratories.

### Immunoprecipitation

Cells were lyzed in IP lysis buffer consisting of 50 mM Tris, pH 8.0, 5 mM EDTA, 150 mM NaCl, 0.5% NP-40, 1 mM phenylmethylsulfonyl fluoride, 1 mM sodium vanadate, 10 mM NaF, Complete Mini Protease Inhibitors (Roche, Nutley, NJ, USA and) and 1 mM PR-619 DUB inhibitor (Calbiochem), at 4°C for 30 min. Supernatants were then incubated for 4 h with HA Epitope Tag Antibody, Agarose conjugate (2-2.2.14) under constant rotation at 4°C. Beads were washed five times in ice-cold lysis buffer and eluted protein was subjected to SDS-PAGE and immunoblotted with the indicated antibodies.

### Viability assay

Cell viability was assessed 24 h after drug treatment using the CellTiterGlo Luminescent Cell Viability Assay (Promega, Fitchburg, WI, USA) per the manufacturer's instructions. Following compound treatments, CellTiterGlo reagent was added to the cells; after a 10-min incubation period to allow for stabilization of luminescence, samples were transferred to solid white multiwell plates and luminescence was read on a PHERAstar FS microplate reader (BMG LABTECH, Ortenberg, Germany). In all cases, error bars represent SD from triplicate experiments, and asterisks indicate *p* < 0.001 (***) or *p* < 0.01 (**) compared to the respective controls using Student's unpaired *t*-test; ‘ns’ means there was no significant difference.

### qRT-PCR

Total RNA was extracted using RNeasy Mini Kit (Qiagen) and complementary DNA (cDNA) was synthesized using the ImProm-II Reverse Transcription System (Promega). Ten nanograms cDNA was used per PCR reaction with SYBR Green PCR Master Mix (Applied Biosystems, ABI) and quantified on the BIORAD CFX96 Real Time System. Fold changes in mRNA expression was quantified using the ^Δ-Δ^Ct algorithm with *18S* ribosomal RNA as loading control. qPCR primers are tabulated in Supplemental Experimental Procedures.

### Immunofluorescence

U2OS were seeded onto coverslips pre-coated with gelatin. Following RNAi or compound treatments, cells were fixed in 4% paraformaldehyde. Cells were permeabilized with 0.05% Triton X-100 and coverslips were blocked in 10% bovine serum albumin/PBS for 20 min. Cells were incubated with anti-TOMM20 (Santa Cruz Biotechnology) at 1:100 dilution for 1 h to stain mitochondria. Following three washes with PBS, cells were incubated with Alexa-488 (Life Technologies) at a dilution of 1:400 and DAPI (1:3000) for 1 h. Coverslips were washed and mounted with glycerol on glass slides. Imaging was performed on the Leica TCS SP2 AOBS laser confocal scanner mounted on a Leica DM-IRE2 inverted microscope with a 63× oil immersion objective.

### Gene expression and multiple linear regression analyses

We used a classical statistical modeling approach (Multiple Linear Regression with multiple variables) to relate drug sensitivity to the expression profiles of the 8 selected genes. Data for sensitivity to ABT-263 were downloaded from the Wellcome ‘Genomics of Drug Sensitivity in Cancer’ database (http://www.cancerrxgene.org/translation/Drug/1011) and RNA expression data for the corresponding cell lines were retrieved from the ‘Whole Genome Project’ section of the COSMIC public database (http://cancer.sanger.ac.uk/cosmic) of the Welcome Trust Sanger Institute. Further details and raw data can be found in Supplemental Experimental Procedures.

## SUPPLEMENTARY FIGURES AND TABLE




