# Supplementary material for: Inhibition of MARCH5 ubiquitin ligase abrogates MCL1-dependent resistance to BH3 mimetics via NOXA
Source: Oncotarget. 2016 Feb 21;7(13):15986–6002. doi: 10.18632/oncotarget.7558 (PMC4941292; doi:10.18632/oncotarget.7558)
Supplement: Supplementary file 1 [file oncotarget-07-15986-s001.pdf]

Inhibition of MARCH5 ubiquitin ligase abrogates MCL1-dependent resistance to BH3 mimetics *via* NOXA

Supplemental Information

Subramanian et al., Mitochondrial MARCH5 ubiquitin ligase abrogates MCL1-dependent resistance to BH3 mimetics via NOXA

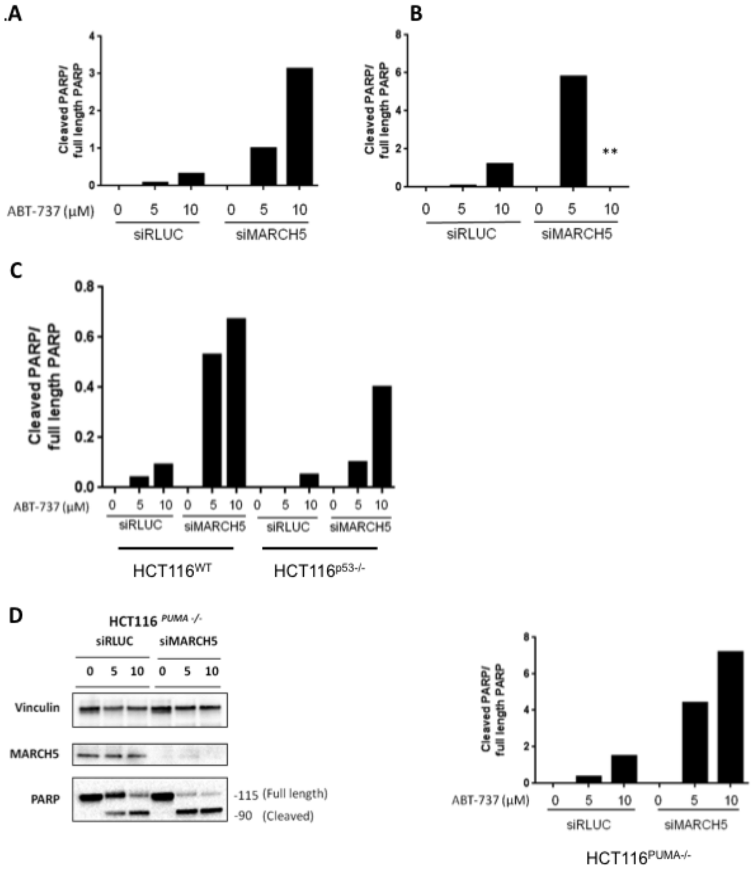

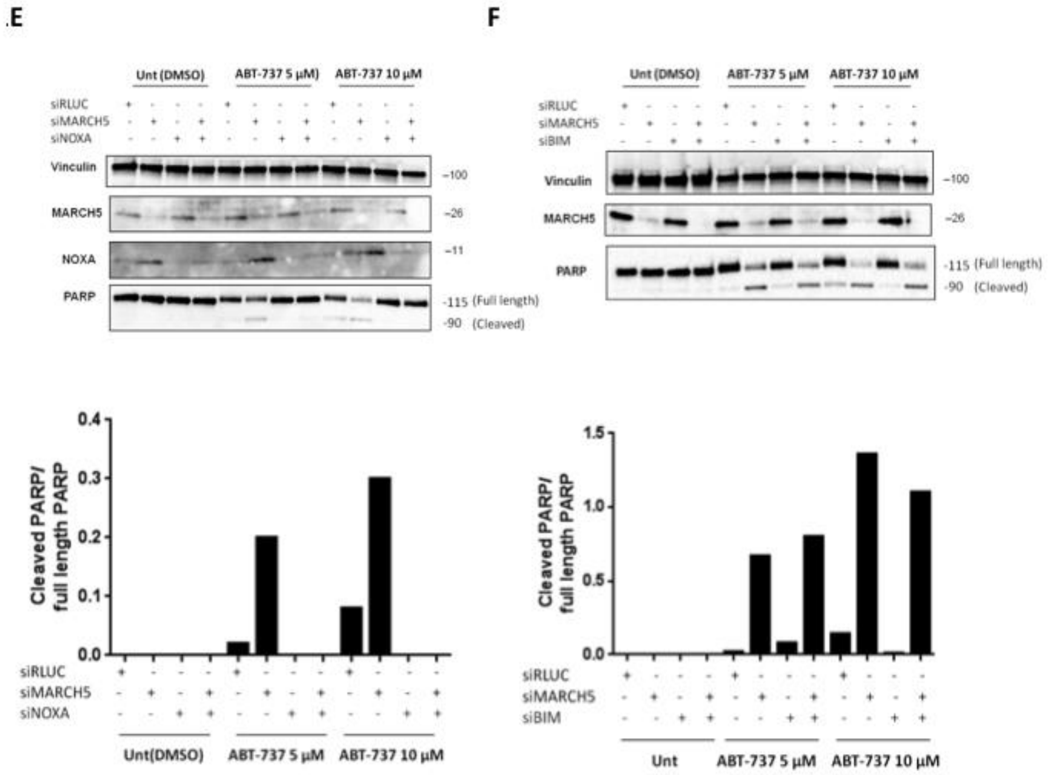

Figure S1, Quantification of PARP cleavage as an indicator of apoptosis. Intensity ratio of cleaved:full-length PARP related to U2OS (A) and HCT116 (B) from Figure 1 is shown; asterisks indicate quantification was not possible due to complete absence of uncleaved PARP. (C) Related to comparison of viability in HCT116 p53 WT and NULL shown in Figure 3B and C. (D) Confirmation that sensitization to ABT-737 does not require PUMA, and that the mode of cell death is apoptosis; left panel is western blot and right panel is quantification of PARP cleavage related to Figure 3D. (E, F) Confirmation that knockdown of NOXA (E) but not BIM (F) abrogates MARCH5-dependent sensitization to ABT-737; panels are related to the viability data shown in Figure 4C and D. BIM knockdown was validated at the mRNA level (Figure S7).

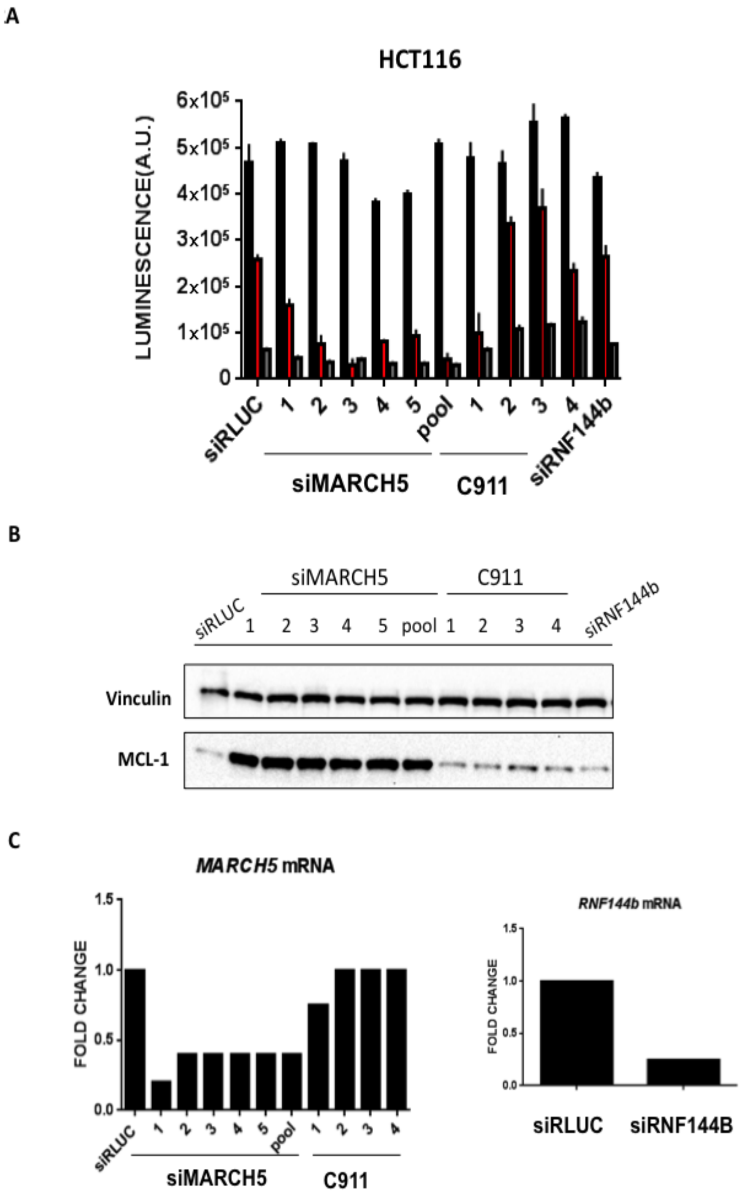

Figure S2, Related to Figure 1; siRNA deconvolution confirms the effects of MARCH5 on sensitization to apoptosis and MCL-1 stabilization are on-target (A) HCT116 cells were transfected with the given siRNAs and treated with ABT-737 at 5 or 10 μM ABT-737 (red and grey bars, respectively). Cell viability was measured using CellTiter-Glo. The error bars represent the SD from triplicate measurements for each condition. (B) Whole cell lysates from HCT116 cells transfected with the indicated siRNAs were subjected to SDS-PAGE and immunoblotted with the given antibodies. (C) Knockdown of *MARCH5* and *RNF144B* mRNA was validated by quantitative RT-PCR. Values were normalized to *18S* mRNA. Sequences of C911 controls (Buehler et al., 2012) are given in Supplemental Experimental Procedures.

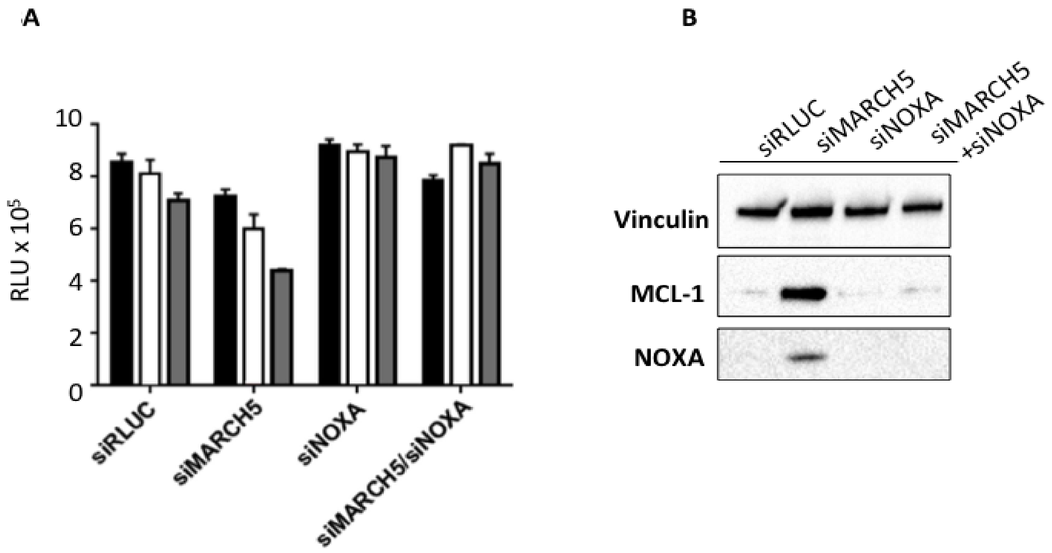

Figure S3, Related to Figure 4; Cross-vendor validation that NOXA is required for MCL1 stabilization and sensitization to ABT-737 after loss of MARCH5. (A) HCT116 cells were transfected with the indicated esiRNAs and treated with DMSO (black bars) or ABT-737 (5 or 10 μM, white or gray bars, respectively) for 24 h. Cell viability was measured using CellTiter-Glo. The error bars represent the SD from triplicate measurements for each condition. (B) Whole cell lysates from HCT116 transfected with the indicated esiRNAs were subjected to SDS-PAGE and immunoblotted with the indicated antibodies.

Table S1, Related to Figure 6 Results of optimized multiple linear regression analysis for all cell lines (n = 648), those with nonfunctional p53 (n = 427), those with wild type p53 (n = 221), and the wild type p53 blood subset. Each row shows the estimate value (i.e., the unit change in IC_{50}) that is predicted for every unit increase in expression of the particular gene. For example for every unit increase in MCL1 in cells with nonfunctional (MUT/DEL) p53, the IC_{50} value is predicted to increase by 1.47 (i.e., cells become less sensitive). SE, standard error of the estimate value. p values indicate whether each gene (or pairwise interaction) was deemed significant (*p < 0.05; **p < 0.01). An empty row indicates that the interaction was not considered significant in the relevant subset. Raw data and further explanation of the steps of the analysis can be found in the Supplementary Information.

A

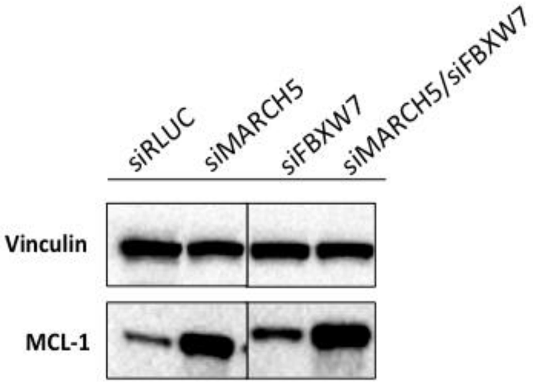

B

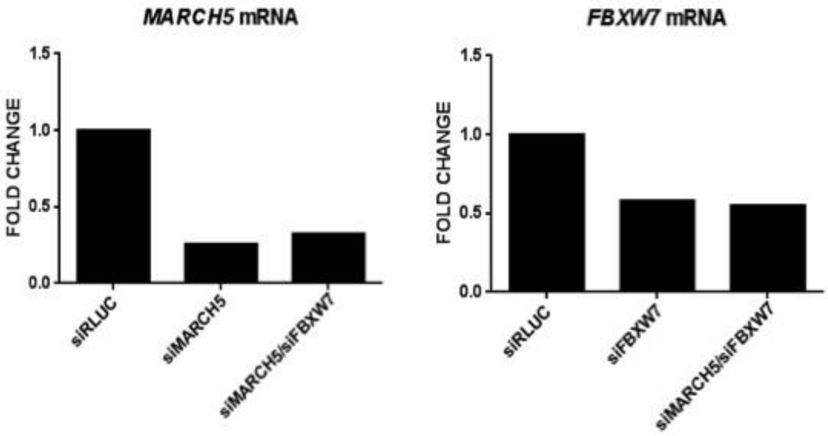

Figure S4 MARCH5-dependent stabilization of MCL-1 occurs independently of FBXW7 (A) Whole cell lysates from HCT116 cells transfected with the indicated siRNAs were subjected to SDS-PAGE and immunoblotted with the indicated antibodies. (B) Knockdown of *MARCH5* and *FBXW7* was assessed through quantitative RT-PCR normalized to *18S* as loading control.

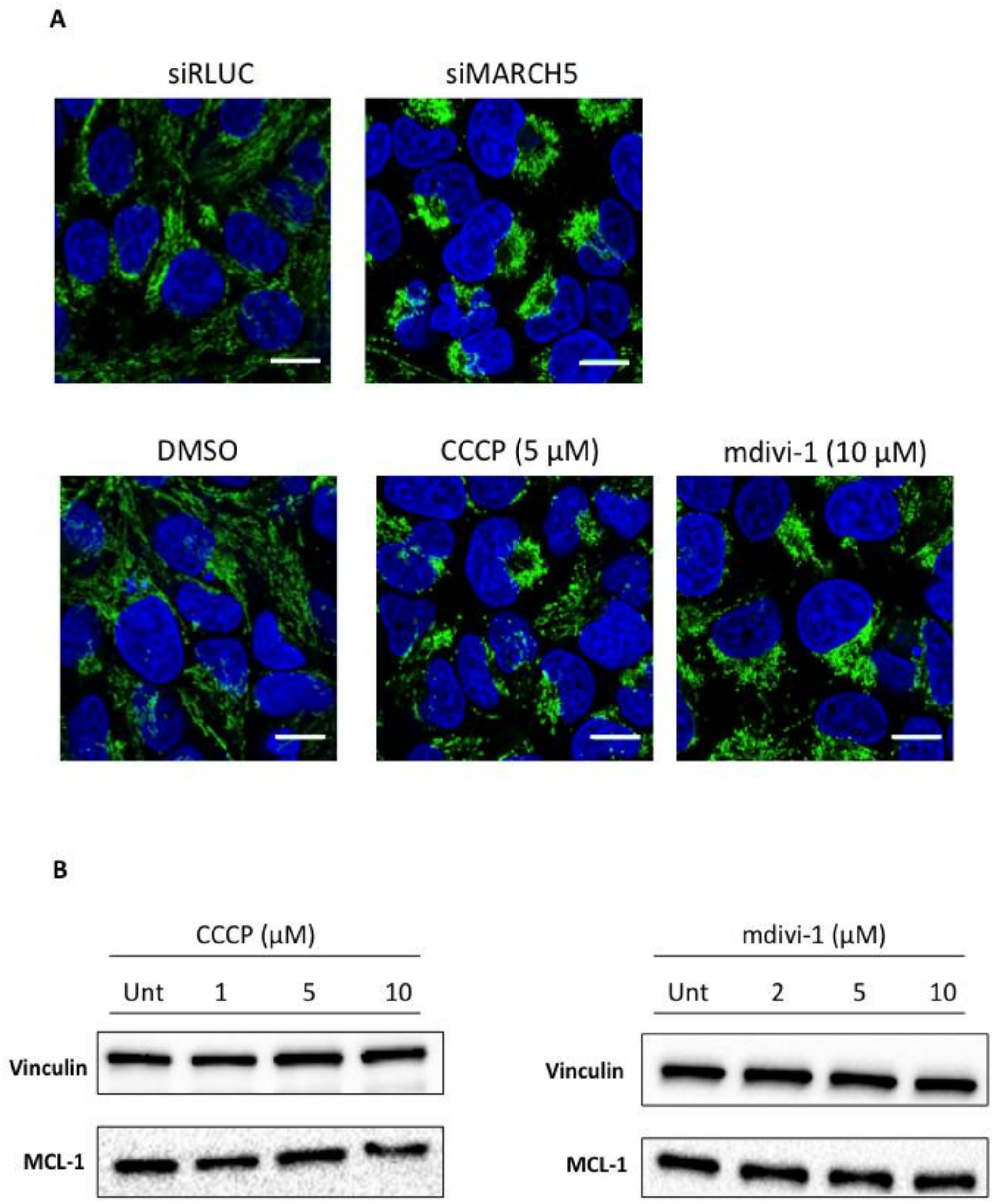

Figure S5 MCL-1 stabilization occurs independent of changes in mitochondrial morphology (A) U2OS cells were plated on coverslips and transfected with the siRLUC, siMARCH5 for 72 h or treated with CCCP (5 μM) for 4 h and mdivi-1 (10 μM) for 48 h. Cells were then fixed in 4% paraformaldehyde and immunostained with anti-TOMM20 to stain mitochondria and DAPI for nuclear staining. Scale bars represent 10 μM. (B) Whole cell lysates from U2OS cells treated with CCCP or mdivi-1 at the indicated concentrations were subjected to SDS-PAGE and immunoblotted with the indicated antibodies.

A

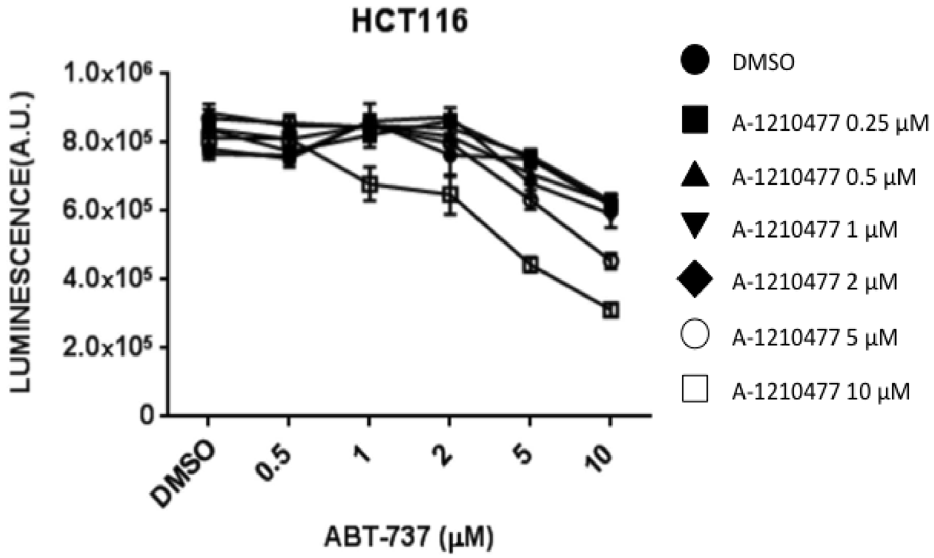

B

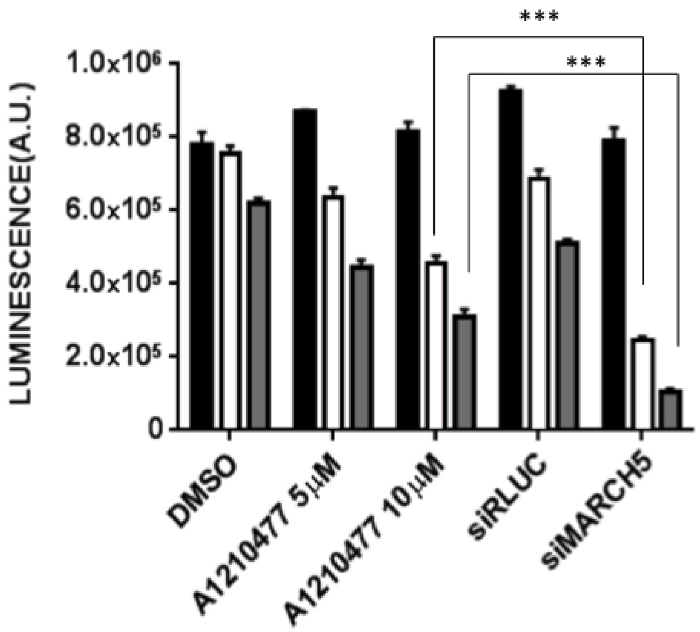

Figure S6, Related to Figure 7; ABT-737 synergizes with high concentrations pf the MCL1-specific inhibitor, A-1210477 (A) HCT116 cells were treated with ABT-737 and A-1210477 either alone or in combination. (B) Both MARCH5 targeting and MCL1 inhibition are effective strategies for sensitization to ABT-737. Cells were treated with either A-1210477, siRLUC, or siMARCH5 prior to treatment with DMSO (black bars) or ABT-737 (5 or 10 μM, white or grey bars, respectively) for 24 h. Cell viability was measured using CellTiter-Glo. Error bars represent the SD of triplicate measurements.

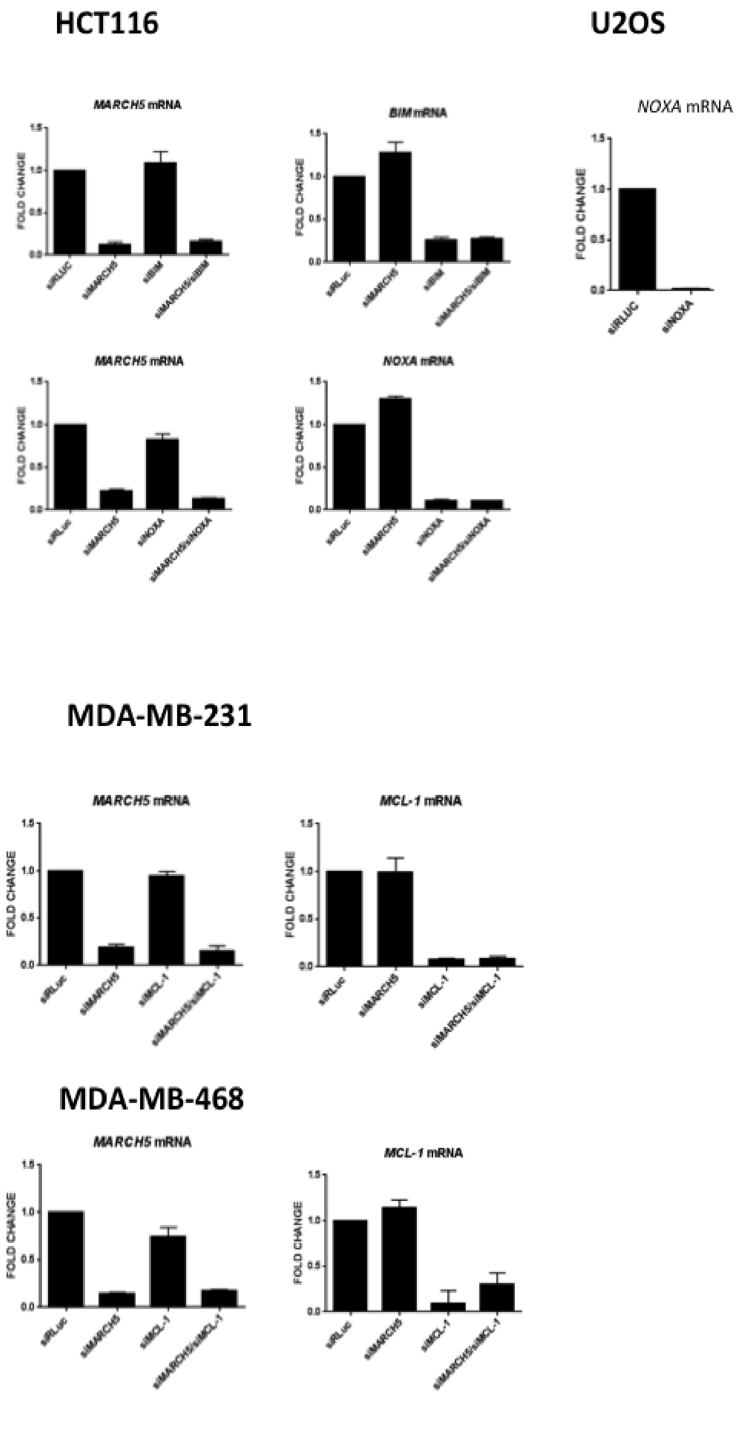

Figure S7, Validation of siRNA-mediated knockdown. Data for HCT116, U2OS, and for MDA-MB-231 and MDA-MB-468 cells are related to Figures 4A and B, Figure 5C, and Figure 7E and F, respectively. Cells were transfected with the indicated siRNAs. Knockdown for the respective genes was assessed by quantitative RT-PCR normalized to *18S* mRNA as loading control.

Supplemental Experimental Procedures

Primers used for qPCR

	Forward (5'-3')	Reverse (5'-3')
18S	GATTAAGTCCCTGCCCTTTGTACA	GATCCGAGGGCCTCACTAAAC
MARCH5	GATGCTGGACAGAAGTTGCTGG	CCACTCTGGCTGTACTGTTTCC
MCL-1	GGTGCCTTTGTGGCCAAACACTTA	ACCCATCCCAGCCTCTTTGTTTGA
PUMA	ACGACCTCAACGCACAGTACG	TCCCATGATGAGATTGTACAGGAC
p21	CTGGAGACTCTCAGGGTCGAAA	GATTAGGGCTTCCTCTTGGAGAA
NOXA	CAGGACTGTTCGTGTTCAGC	TTCTGCCGGAAGTTCAGTTT
RNF144B	CATTATGACAAAGGGCCATGC	CATACATTTTGCTGGTACTGCC
BIM	TGGCAAAGCAACCTTCTGATG	GCAGGCTGCAATTGTCTACCT
FBXW7	CCACTGGGCTTGTACCATGTT	CAGATGTAATTCGGCGTCGTT

siRNA sequences

Gene Symbol	Gene ID	Catalog Number	Sequences
MARCH5	54708	M-007001-01	UCAAACAGCAGCAAUAUUU<br>GGACAGCUGUGACUUAUGG<br>GUAAAUUGAUGUUCAGUAG<br>GACAGAAGUUGCUGGGUUU
MARCH5 C911 Controls	n/a	Custom	GUAAAUUGAUGUUCAGUAG<br>GCUGAAUACCUAAUAGUUU<br>GCGCAAAUACUCGAAUAAA<br>GAAUAAUGGUCGGCUCUAU
MCL1	4170	M-004501-008	CGAAGGAAGUAUCGAAUUU<br>AGAACGAAUUGAUGUGUAA<br>GGACCAACUACAAAUUAAU<br>GCUACGUAGUUCGGGCAAA
BIM	10018	M-004383-02	CCGAGAAGGUAGACAAUUG<br>UGAUGUAAGUUCUGAGUGU<br>AUGUAAGUUCUGAGUGUGA<br>GUUCUGAGUGUGACCGAGA
NOXA	5366	M-005725-03	AAACUGAACUUCCGGCAGA<br>AAUCUGAUAUCCAAACUCU<br>CUGGAAGUCGAGUGUGCUA<br>GCAAGAACGCUCAACCGAG
PLK1	5347	M-003290-01	CAACCAAAGUCGAAUAUGA<br>CAAGAAGAAUGAAUACAGU<br>GAAGAUGUCCAUGGAAAUA<br>CAACACGCCUCAUCCUCUA
RNF144B	255488	M-025119-01	CAGCUUGCCUGAAACAGUA<br>AAGCUGAGAUUGCCUGUUU<br>GUAGAGACAGUCAGCCUAU<br>GGGUUUAUAUCGAACGCAA

Gene expression and multivariate analysis (related to Figure 6)

Together with MARCH5, we selected BAX and BAK (since they are terminal effectors of the response to ABT-737 and ABT-263), NOXA and BCL2L1/BCLXL (based on our current data), BCL2L11/BIM (as it is implicated in the response to ABT-737 and ABT-263), and MCL1 (a well-documented determinant of sensitivity to ABT-737 and ABT-263). We also included HUWE1, an MCL1 ubiquitin ligase that can accelerate BH3-dependent apoptosis. For 648 of the 971 cell lines in the database, both ABT sensitivity and mRNA expression data (for *MARCH5*, *MCL1*, *BIM*, *BAX*, *HUWE1*, *BAK1*, *NOXA*, and *BCLXL*) were available. The RNA expression levels are all normalized (z-score) in order to standardize gene expression estimates obtained different platforms used for sequencing the samples (IlluminaHiSeq_RNASeqV2, IlluminaGA_RNASeqV2, AgilentG4502A_07_3). For the sensitivity parameter in our models, we used IC_{50} values for ABT-263 for each cell line. For some analyses, we

further subdivided the dataset into cell lines with wild type p53 (221 samples) and those with mutant/deleted p53 (427 samples). For optimization of the model, we used the Akaike Information Criterion (AIC). The model optimization was done in a backward selection fashion, starting from the full linear model specification, (i.e., mRNA expression levels for each individual gene, and for all possible pairwise combinations were provided prior to optimization). Optimization was run separately for each individual subset (all wild type p53 samples, all p53 mutant/deletion samples (MUT/DEL), or blood wild type p53 samples). See Table S1 for all estimates of effect sizes and associated p values for the wild type p53, mutant p53, and wild type p53 blood subsets.

Supplemental References

Buehler, E., Chen, Y.C., and Martin, S. (2012). C911: A bench-level control for sequence specific siRNA off-target effects. PLoS One *7*, e51942.
